# Use of the ODD-Luciferase Transgene for the Non-Invasive Imaging of Spontaneous Tumors in Mice

**DOI:** 10.1371/journal.pone.0018269

**Published:** 2011-03-29

**Authors:** Scott J. Goldman, Elizabeth Chen, Robert Taylor, Sheng Zhang, Whitney Petrosky, Michael Reiss, Shengkan Jin

**Affiliations:** 1 Department of Pharmacology, University of Medicine and Dentistry of New Jersey, Robert Wood Johnson Medical School, Piscataway, New Jersey, United States of America; 2 Department of Medicine, University of Medicine and Dentistry of New Jersey, Robert Wood Johnson Medical School, New Brunswick, New Jersey, United States of America; 3 Department of Oral and Maxillofacial Surgery, Second Xiangya Hospital, Central South University, Changsha,China; 4 Cancer Institute of New Jersey (CINJ), New Brunswick, New Jersey, United States of America; Florida International University, United States of America

## Abstract

**Background:**

In humans, imaging of tumors provides rapid, accurate assessment of tumor growth and location. In laboratory animals, however, the imaging of spontaneously occurring tumors continues to pose many technical and logistical problems. Recently a mouse model was generated in which a chimeric protein consisting of HIF-1α oxygen-dependent degradation domain (ODD) fused to luciferase was ubiquitously expressed in all tissues. Hypoxic stress leads to the accumulation of ODD-luciferase in the tissues of this mouse model which can be identified by non-invasive bioluminescence measurement. Since solid tumors often contain hypoxic regions, we performed proof-of-principle experiments testing whether this transgenic mouse model may be used as a universal platform for non-invasive imaging analysis of spontaneous solid tumors.

**Methods and Materials:**

*ODD-luciferase* transgenic mice were bred with *MMTV-neu/beclin1+/−* mice. Upon injection of luciferin, bioluminescent background of normal tissues in the transgenic mice and bioluminescent signals from spontaneously mammary carcinomas were measured non-invasively with an IVIS Spectrum imaging station. Tumor volumes were measured manually and the histology of tumor tissues was analyzed.

**Conclusion:**

Our results show that spontaneous mammary tumors in ODD-luciferase transgenic mice generate substantial bioluminescent signals, which are clearly discernable from background tissue luminescence. Moreover, we demonstrate a strong quantitative correlation between the bioluminescent tumor contour and the volume of palpable tumors. We further demonstrate that shrinkage of the volume of spontaneous tumors in response to chemotherapeutic treatment can be determined quantitatively using this system. Finally, we show that the growth and development of spontaneous tumors can be monitored longitudinally over several weeks. Thus, our results suggest that this model could potentially provide a practical, reliable, and cost-effective non-invasive quantitative method for imaging spontaneous solid tumors in mice.

## Introduction

In human medicine, the detection and evaluation of tumors has been an area of ongoing research for many years. The advent of radiology as a diagnostic aid provided the first opportunity to image internal tumors non-invasively, and progress over the past three decades has resulted in a wide array of non-invasive, live-tissue imaging options [1], [2], [3], [4]. Examples of technology in this rapidly expanding field include x-rays, ultrasonic imaging, computed tomography, positron emission tomography, magnetic resonance imaging, and nuclear scintigraphy. Many of these imaging modalities can provide astonishingly detailed information about a tumor, including size, shape, location, vasculature and architecture; but most of the advanced techniques require extensive training and experience to master, hence the existence of board-certified radiologists within the medical profession.

In the laboratory, tumor imaging is a very different process. The evolution of animal models for tumor research has resulted in the predominant use of immunocompromised mice as hosts for human xenograft and mouse allograft tumors, allowing researchers to observe the growth and development of tumors in a “living test tube.” While these models have made tremendous contributions to our knowledge in the field of oncology, they also possess inherent drawbacks which limit their usefulness [5], [6]. First and foremost, it has long been known that xenografts/allografts in mice do not necessarily mimic the behavior they exhibit in their native microenvironment [6], [7], [8]. Furthermore, the mice used in these models are invariably immunocompromised to prevent rejection of the xenograft. This condition alters the natural inhibitory factors that cancer is subjected to in an otherwise immunocompetent host [6], [7], allowing the xenograft to grow and even metastasize. In the meantime, it also abolishes the pro-tumorigenic inflammatory responses that are relevant to the natural evolution of tumors [9].

The recognition of the deficiencies of xenograft/allograft models has led to a number of efforts to develop better mouse tumor models, imaging systems, or both. With the recent advances in genetic manipulation in mice, we are now able to selectively knock out a tumor suppressor gene or overexpress an oncogene which results in the spontaneous development of tumors in specific tissues [6], [10], [11]. These models, of course, are not perfect, and mouse tumors do not always behave similarly to their human counterparts. In many instances, however, these spontaneous murine tumor models can closely mirror human tumors with the same genetic alterations. They are also excellent animal model systems for determining the efficacy of anti-cancer therapeutics.

Although these genetically modified mice have provided us with a superior model of spontaneous tumor development, the non-invasive imaging of these tumors continues to pose a problem. Imaging methods that are common in human oncology such as ultrasonography, computed tomography, and magnetic resonance imaging are often impractical for use in the laboratory due to expense, safety, and the need for extensive technical expertise. The most popular alternative has been the pursuit of bioluminescent or fluorescent imaging techniques. In recent years, a number of effective *in vivo* imaging models have been developed which depend either upon the conditional expression of bioluminescent reporter genes in concert with genes associated with a certain type of tumor, or upon the use of fluorescent reporter molecules directed towards specific cancer-associated cell proteins [12]. The limitations of these models is that they are specific for only a single type or subtype of cancer, and may not be broadly applied to tumors of differing origins.

**Figure 1:**
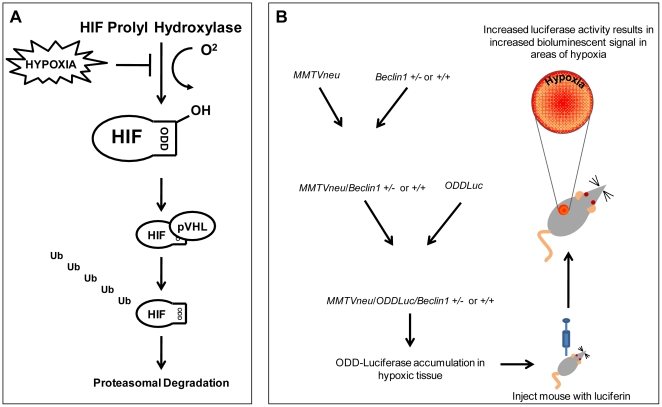
The effect of hypoxia on the HIF1-α degradation pathway and the scheme of the use of the *ODD-Luciferase* transgene for bioluminescent imaging of spontaneous tumors. (A) HIF prolyl hydroxylase depends upon the substrate oxygen for hydroxylation of the ODD domain in constitutively expressed HIF1-α protein in normoxic cells, ultimately leading to the degradation of HIF1-α via the ubiquitin-proteasome pathway. The expression of the *ODD-Luciferase* transgene is identically regulated, leading to the accumulation of the ODD-Luciferase protein under hypoxic conditions. (B) A schematic of the generation of mammary carcinoma-prone *MMTV-neu/ODD-Luc/Beclin1 +/−* mice.

The core of developing any universally applicable tumor imaging system is the differentiation of tumor cells from normal tissue. At the physiological and metabolic levels there are differences between cancerous tissue and normal tissue that, if exploited, might provide a foundation for a more broadly applicable tumor imaging system [13]. It has long been known that despite their efforts to encourage angiogenesis, solid tumors experience a certain degree of relative hypoxia when compared with surrounding tissue [14], [15], [16]. Recently, a mouse model has been developed in which regions of hypoxia may be imaged via bioluminescent imaging [17]. In this model, Safran, *et al.* created a transgenic mouse expressing the oxygen-dependent degradation (ODD) domain of the Hypoxia Inducible Factor 1-α (HIF1-α) gene fused to a luciferase bioluminescent reporter gene. The HIF1-α gene is ubiquitously transcribed and translated, but under normoxic conditions, the enzyme HIF prolyl hydroxylase utilizes oxygen to hydroxylate the HIF ODD. The hydroxylated ODD then recruits von Hipple-Lindau protein (pVHL), a ubiquitin E3 ligase, leading to poly-ubiquitination of HIF1-α and its subsequent degradation by the proteasome (Fig. 1A). Conversely, under hypoxic conditions, lack of hydroxylation of the ODD domain leads to the accumulation of HIF1-α which acts as a transcriptional regulator for a number of hypoxia-induced genes. By universally expressing the ODD-luciferase gene in mice, Safran, *et al.* developed an organism in which luciferase is continuously transcribed and translated in all cells, but is also be rapidly degraded under normoxic conditions. Only under conditions of hypoxia does luciferase accumulate and thus, by providing the substrate luciferin, regions of hypoxia within these mice may be imaged with bioluminescent imaging equipment.

We reasoned that solid tumors should be visible in ODD-luciferase expressing mice due to the relative hypoxia experienced by the tumor cells and hypothesized that the *ODD-luciferase* transgenic model could serve as a universal platform for non-invasive imaging of spontaneous solid tumors in mice. In this report we conducted proof-of-principle studies by cross-breeding transgenic mouse strains known to be predisposed to the development of mammary gland tumors, the *MMTV-neu* [18] and *Beclin1 +/−* [19] mouse strains, with the *ODD-luciferase* mice developed by Safran, *et al.* and subsequently characterized tumors via bioluminescent monitoring. Our results show that mammary tumors which develop in ODD-luciferase transgenic mice generate substantial bioluminescent signals which are clearly discernable from background tissue luminescence. Furthermore, we were able to clearly track tumor growth and development longitudinally over several weeks and were able to observe changes in tumor size in response to chemotherapeutic treatment using this system. Thus, our study provides experimental evidence supporting the principle that this system may have potential for providing a universal model for the non-invasive monitoring of spontaneously occurring solid tumors in mice.

## Results

### Generation of ODD-luciferase transgenic mice predisposed to mammary tumors and determination of baseline organism-wide bioluminescent signals associated with relative hypoxia

Early imaging data on *ODD-Luc* mice was obtained by Safran, *et al.* in their description of this model [17]. Our initial objective was to determine whether or not *ODD-Luc* transgenic mice could be successfully interbred with other, non-*ODD-Luc* transgenic mice to produce tumorigenic offspring capable of being imaged with available bioluminescence detection technology. Specifically, we sought to exploit the principle of tumor hypoxia as a means of monitoring tumor growth and response to treatment in populations of wild-type and reduced-autophagy mice. In our laboratory, we maintain a colony of mice heterozygous for the haploinsufficient tumor suppressor gene *beclin1* [*19*], an autophagy-related gene commonly deleted in human breast cancers. In an effort to generate mammary tumors with *beclin1* deletion, these mice were crossed with mice bearing the *MMTV* (mouse mammary tumor virus promoter)-*neu* transgene. Mutation of the human *neu* homologue, the *her2* oncogene, is associated with the development of certain human breast tumors [20], [21], [22], [23]. The resulting *MMTV-neu/beclin1+/+ or +/−* mice were further crossed with *ODD-luciferase mice*. As expected, offspring develop mammary gland tumors with high frequency (Fig. 1B, Table S1).

**Figure 2:**
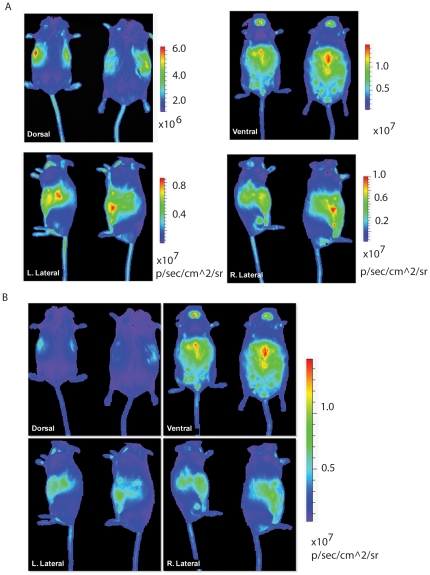
Evaluation of baseline bioluminescent signals in *MMTV-neu/ODD-Luc/Beclin1 +/+ or +/− mice*. (A) Representative dorsal, ventral, right lateral, and left lateral views of two female mice expressing the *ODD-Luc* and *MMTV-neu* transgenes. Each image is individually scaled for radiance according to scale bars shown. (B) The images in panel (A) are adjusted to conform to the same radiance scale, showing the relative intensity of signaling among all images.

Prior to tumor development, we analyzed the baseline bioluminescence in these tumorigenic mice. Figure 2 shows representative images of normal, tumor-free (as determined by post-imaging necropsy), aged (18 month old) female mice. Images were obtained of four views: dorsal, ventral, right lateral, and left lateral (Fig. 2A), with the radiance for each image independently scaled. Radiance is defined as photons/second/cm^2^/steradian and is considered the rate at which any given point in the image generates photons. This rate is commonly used to compare bioluminescent signals among different images taken in different subjects or at different times, as it eliminates much of the variability inherent in comparing raw photon “counts” for a given area for a given image. In each view we observed a unique signal distribution correlating to hypoxic area nearest the camera lens. In the dorsal view, the kidneys generate a marked signal, while in the ventral view, the abdominal fat pad and teats generate relatively high background signals over the abdomen and the thyroid generates a high intensity signal in the cervical region. The lateral views each contain high intensity regions that correlate with the respective kidney for that side, as well as signals generated by the abdominal fat pad and the thyroid (Fig. 2A). Later, the images were merged and forced to conform to a common scale, (Fig. 2B), providing an accurate comparison of relative hypoxia among views and revealing that the ventrum was consistently the region with the highest background signal. Finally, a variety of areas were observed to undergo transient or shifting hypoxia, including the ears, tail, rump, feet, and scruff. These areas can become transiently hypoxic in association with a variety of factors, including changes in ambient temperature, length of time under anesthesia, and sometimes the degree of strength with which the mice were scruffed for restraint prior to luciferin injection. However, it is clear that the bioluminescence in these regions is low and transient when compared to the major hypoxic regions of the kidneys, ventral abdomen, and thyroid gland.

### Analysis of spontaneously developing mammary tumors in ODD-luc transgenic mice

**Figure 3:**
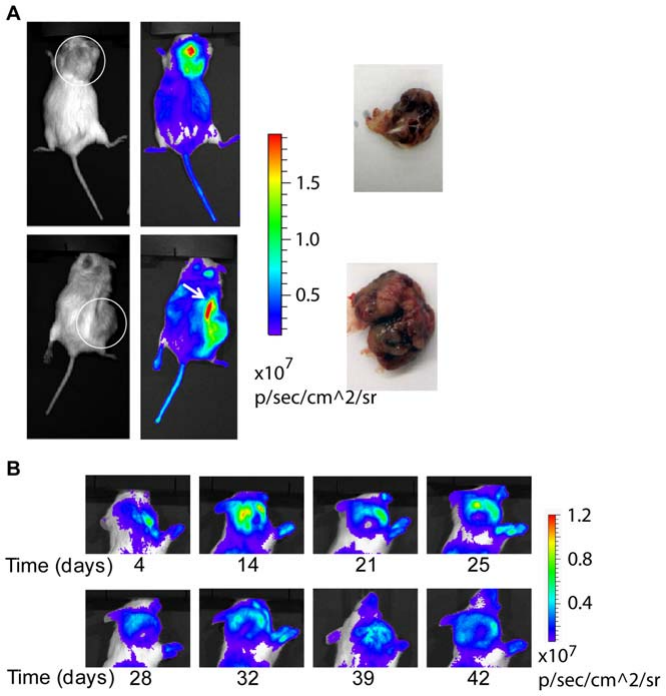
Bioluminescent images of spontaneous tumors. (A) Photographic and luminescent images of two female mice, one bearing a subcutaneous tumor above the right scapula (top panels) and another bearing a subcutaneous tumor on the right abdomen/inguinal region (bottom panels) with photographs of the dissected tumor masses. White circles denote the approximate boundaries of the tumor masses in this view. Note the underlying strong signal generated by the right kidney (arrow) intensified due to decreased subcutaneous and adipose tissue mass. (B) Longitudinal tracking of subcutaneous tumor development in a female mouse.

Subcutaneous tumors began to appear on *MMTV-neu/ODD-luc/Beclin1* mutant mice at approximately 11 months of age, regardless of *beclin1* deletion status (Table S1). Initial imaging candidates were selected and, when the tumors had reached approximately 1 cm in diameter, the mice were subjected to imaging and subsequently sacrificed. Necropsy was performed for acquisition of gross and histological pathology data. As shown in Figure 3A, the imaging sessions revealed obvious hypoxic signatures that correlated with the observable tumor shape and size. One subject (upper panels) bore a spherical mammary carcinoma over the right shoulder generating a substantial bioluminescent signal sufficient to suppress the normally significant background signal generated by the kidneys. Another subject (bottom panels) possessed a multilocular mammary carcinoma located in the right ventral abdomen. Unlike the subject in the upper panels, the tumor in this mouse generated a signal similar in intensity to the right kidney, as evidenced by the hypoxic zone noted just cranial to the tumor signal (Fig. 3A, arrow). In both cases, tumors were clearly identifiable as areas of relative hypoxia when compared to their surrounding, normoxic, tissues.

In addition, we monitored palpable tumors over a six-week period in several mice. Tumor contour was clearly visible on bioluminescent imaging and changes in tumor size, shape, and regions of hypoxia were observed. The imaging signature for tumors consisted of a peripheral region areas of high signal intensity surrounding a central region of low signal intensity. The intensity of luciferase signals at the specific peripheral areas varied among tumors and also varied within tumors at different time points, but the distribution pattern and the collective contour or outline of the high-intensity signaling always correlated with visible tumor boundaries (Fig. 3B).

### Histological analysis of ODD-luc/MMTV-neu mammary carcinoma and the expression pattern of ODD-Luciferase

**Figure 4:**
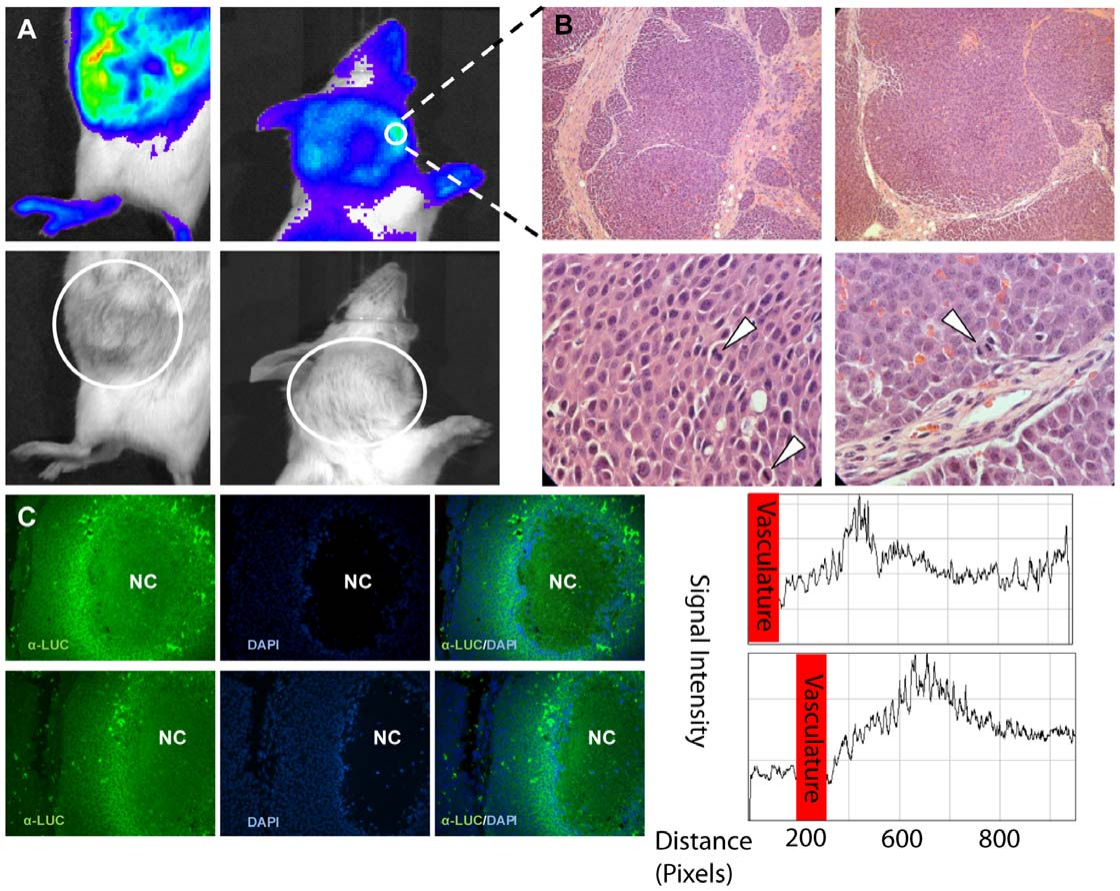
Histological analysis of mammary carcinomas and the expression of ODD-luciferase. (A) Representative subcutaneous tumors on the left cervical and right abdominal aspects of a female *MMTV-neu/ODD-Luc/Beclin1 +/−* mouse. White circles denote approximate boundaries of tumor masses. (B) Hematoxylin and Eosin stained paraffin-embedded tumor tissue sections at 200× (top panels) and 1000× (bottom panels) magnification. White arrows denote the presence of mitotic cells in the viable peripheral tumor tissue. (C) Immunofluorescent analysis of two tumor tissue samples with anti-luciferase antibody. Left panels show immunofluorescent signaling representing the presence of luciferase. Center panels are DAPI stained representing the nuclei of viable cells. Right panels are merged fluorescein/DAPI images. NC: necrotic core (region without DAPI staining). Histograms illustrate the fluorescein signal intensity in relation to the distance from local vasculature.

The heterogeneous nature of the signals observed in these spontaneous tumors suggested that the tumors were also heterogeneous and the different areas of the same tumor might experience varying levels of hypoxia (Fig. 4A). In an effort to correlate the observed signal patterns with tumor anatomy and physiology, all imaged tumors were dissected during necropsy following the conclusion of the imaging time course and were submitted for histological processing and analysis. Histological slides revealed that observed tumors were multilocular in nature, each possessing multiple small discrete nodules (Fig. 4B, C). Each nodule in a given tumor was made of layers of viable cells surrounding a core of dead, necrotic tissue as evidenced by the lack of DAPI-stained nuclei (Fig. 4A, C). The peripheral areas were found to generate high- intensity bioluminescent signals. When these peripheral tissue samples were subjected to immunofluorescent staining with anti-luciferase primary antibody, the resulting fluorescein signal clearly illustrates an increase in ODD-Luciferase expression in living cells as distance from the available blood supply increases, with a precipitous drop in ODD-Luciferase expression in the necrotic core of the nodule (Fig. 4C). Furthermore, low signal intensity was observed in the central nodules of these tumors (Figure 4A). This is likely attributable to a combination of increased necrotic tissue, which is incapable of generating a signal due to a lack of viable cells containing the necessary ODD-luciferase enzyme, and to decreased vascular supply, which limits the delivery of the substrate luciferin to the tissues located in at the center region of a tumor.

### Tumor-related bioluminescent signal contour, but not radiance, correlates with tumor size

**Figure 5:**
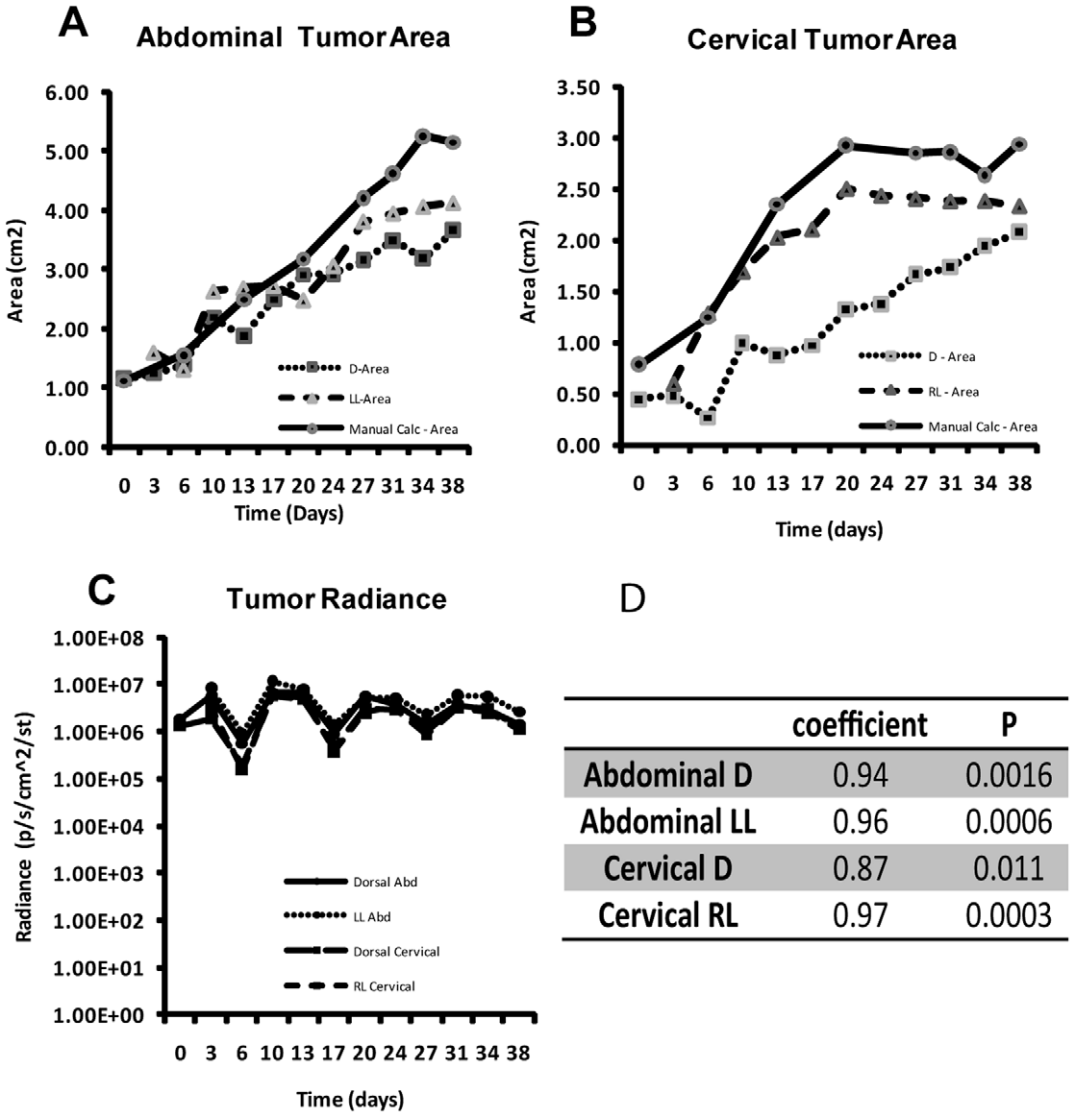
Correlation of tumor bioluminescence signal and tumor volume. (A) Manually calculated tumor area compared to computer-obtained tumor bioluminescent contour by view (left lateral, or LL, versus dorsal, or D) of subcutaneous abdominal tumor in a female *MMTV-neu/ODD-Luc/Beclin1 +/−* mouse over time. (B) Manually calculated tumor area compared to computer-obtained tumor bioluminescent contour by view (right lateral, or RL, versus dorsal, or D) of subcutaneous cervical tumor in the same female *MMTV-neu/ODD-Luc/Beclin1 +/−* mouse over time. (C) Computer-obtained radiance (photons/second/cm^2^/steradian) in both cervical and abdominal tumors over time, by view (right or left lateral and dorsal). (D). Statistical analyses of results from (A) and (B) to determine the correlation between the tumor size measured manually and by bioluminescent signal contour. Pearson coefficients and P values are shown.

We initially expected to see a correlation between overall tumor radiance and tumor size, but quickly realized that three factors complicated this relationship. As previously noted, the tumors being imaged in this study were all heterogeneous in nature, thus resulting in non-uniform levels of ODD-Luciferase expression and activity (Fig. 4A–B). Second, the phenomena of cycling hypoxia [24], [25] led us to believe that the radiance signals from any given tumor could fluctuate over time. Finally, the existence of necrotic tissue and restricted blood flow in the center of growing tumors meant that the relative volume of hypoxic tissue is not proportional to the total tumor volume. In fact, our observations showed that there was remarkably little difference in tumor radiance over nearly five weeks of observation, despite a significant increase in tumor size (Fig. 5C). The variance observed in tumor radiance was remarkably consistent both between tumors and also between views, indicating that the changes were likely related to the kinetics of luciferin injection and uptake, rather than to an actual change in the hypoxic area of the tumors (Fig. 5C). However, by utilizing the region of interest (ROI) tool in the Living Image software suite, we could clearly identify the perimeter of a tumor in a given view based on the peripheral high-signal areas of bioluminescence and obtain a calculated area from that view (Fig. 5A–B). When compared to manually obtained measurements of area for each palpable tumor, the software-obtained areas of tumor bioluminescent contour correlated closely and provided an accurate determination of tumor cross-sectional area (Fig. 5A, B, D). It is important to note that imaging position had an effect on accuracy in determining tumor cross-sectional area, and that the best values were obtained by imaging the mice with the tumor facing directly towards the lens, as expected (Fig. 5B, D).

### Regression of tumors in response to drug treatment can be tracked via bioluminescent imaging

**Figure 6:**
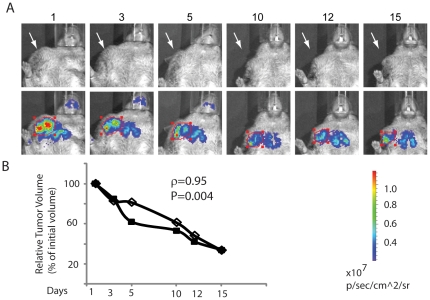
Tracking the regression of spontaneous tumors during drug treatment. (A) Photographic and bioluminescent images of a shrinking palpable tumor over a 14- day drug treatment as described in materials and methods. White arrow in photographic view indicates location of tumor. The red circles in bioluminescent view indicate region of interest. (B) Relative tumor volume expressed as a percent of initial tumor from manually measuring tumor volume (▪) vs. software calculated (ROI) tumor volume (◊) based on bioluminescence signal contour over the course of drug treatment as described in materials and methods. Pearson coefficient (ρ) and P value are shown.

We then performed a proof of principle study to determine if the new spontaneous tumor model could be used for non-invasive *in vivo* imaging of tumor size in response to chemotherapeutics. As shown in Figure 6A, a mouse bearing a palpable tumor was treated with doxorubicin and prednisone for a course of two weeks. As witnessed in both the photographic and bioluminescent images, the palpable tumor decreased in size during the drug treatment. To gain a more quantitative measurement of tumor regression, the tumor dimensions were obtained manually by the use of calipers while the measurement of the bioluminescent region of interest (red circles) was obtained through the Living Image software from the ventral and lateral views. The tumor volume was then calculated based on these measurements as described in material and methods. As shown in Figure 6B, the regression of the tumor volume as measured by bioluminescence signal contour correlates with that determined manually. These results indicate that this imaging system can be used as a technique to track the regression of spontaneous tumors during drug treatment.

### Nascent impalpable mammary tumors are detectable and may be tracked longitudinally in MMTV-neu/ODD-Luc/Beclin1 +/+ or +/− mice via bioluminescent imaging

**Figure 7:**
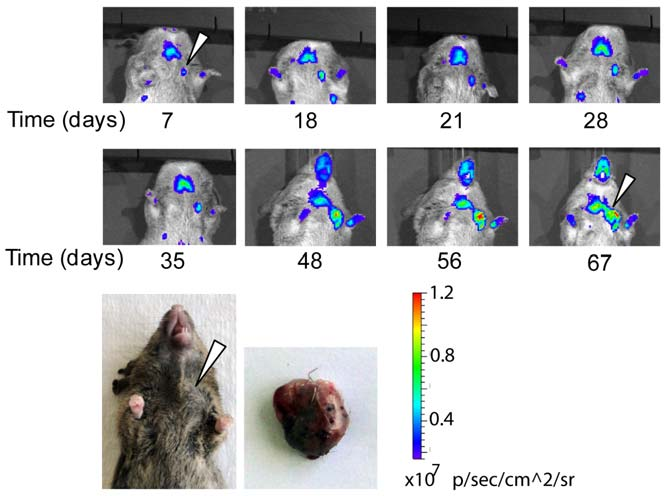
Longitudinally tracking impalpable spontaneous tumors. Left panels: Bioluminescent images of a subcutaneous tumor of the left cranial thoracic mammary gland developing in an agouti female *MMTV-neu/ODD-Luc/Beclin1 +/+* mouse over an eight week period. Arrow indicates tumor signal at beginning and end of imaging period. Only this region persistently shows hypoxic signal throughout the imaging period (the hypoxic signals are expected near the thyroid and the extremities). Note the decrease in background signal observed in agouti mice. This is due to attenuation of the luciferase signal by the darker hair. Right panels: Photograph of external tumor appearance at day 67 of imaging and dissected tumor mass. Arrows denote tumor location.

In the early phase of this study, only mice bearing palpable tumors were imaged. This work established that tumors could be successfully monitored over time for changes in size, shape, and hypoxic regions. Following this finding, we scanned large numbers of mice on a weekly basis to determine at what stage tumors could be imaged. Existing literature has shown that bioluminescent xenografts can be imaged to the single-cell level in nude mice [26] but our system relies upon being able to separate the signal of a hypoxic tumor from the background bioluminescence previously described. We found that this system was capable of detecting tumors in mice at least several weeks before they were palpable. Figure 7 illustrates the continuous tracking of bioluminescent signal in the region of the left cranial mammary gland over a period of 67 days. As expected, the thyroid region and the extremities show hypoxic signals. Starting from day 7, a hypoxic signal (indicated by an arrow, Figure 7) is noticeable and it persists throughout the whole experimental period. The tumor corresponding to the hypoxic signal only became palpable on day 48. These data indicate that this imaging model may serve as a sensitive technique for the detection of the development of very small subcutaneous tumors in haired mice.

## Discussion

Xenografts have served as a valuable model system for cancer biology for many years, but they are subject to significant limitations. While they provide relatively easy imaging of cancer growth, they place the tumor in a non-native, immunocompromised environment that fails to recreate its original *in situ* milieu. The advent of genetic manipulation has led to the development of improved mouse models of cancer in which tumors appear spontaneously and develop naturally in their native environment. These genetically driven models, while superior in providing a more accurate reflection of natural tumor development and behavior, are difficult to image in the laboratory using existing techniques and technology. Here we report a system that could be used as a potentially universal platform for the non-invasive imaging of spontaneously-occurring, solid tumors. Using the *ODD-luciferase* transgenic mouse in concert with tumorigenic mouse mutants, we have analyzed base-line background signals from non-tumor-bearing mice, imaged a series of spontaneously developing mammary tumors over time, and conducted a proof-of principle study showing the feasibility of this model.

Our results indicate that bioluminescent imaging of tumor-bearing *ODD-luciferase* mice can detect mammary tumors. Furthermore, by using the contour of the luminescent signal, we can accurately track tumor growth over time. In particular, this approach can track shrinkage of tumor in vivo in response to cancer chemotherapy. Finally, we have shown that this model is sensitive enough to allow detection of tumors many weeks before they become physically palpable. Taken together, these proof-of-principle findings support that this imaging model is suitable for the non-invasive monitoring of spontaneous mammary tumors in mice.

One important conclusion from our study is that the bioluminescent signal contour rather than the bioluminescent radiance correlates with tumor size. The bioluminescent signal intensity is determined by two major factors, the accumulation of luciferase and the availability of the substrate luciferin. Luciferase accumulates in living but hypoxic cells. Lack of vascularization in mammary tumors on the one hand creates the hypoxic cellular environment which promotes the accumulation of the ODD-luciferase fusion protein; on the other hand, it leads to necrotic cell death at the center of the tumors (Figure 4C). As the tumor volume increases, so does the tumor necrotic center. Consequently the net accumulation of luciferase does not correlate with the tumor volume (Figure 5C). In addition, lack of vascularization greatly reduces the availability of the substrate, luciferin, in the center region of a tumor. This is likely another major reason why the center region of a tumor has low level of bioluminescence intensity and the bioluminescent radiance does not correlate with tumor volume. In contrast, the cells in the peripheral regions of the tumors that undergo normoxia/hypoxia transition (Figure 4) are alive. They experience certain levels of hypoxic stress and have access to the substrate luciferin. Thus these cells consistently exhibit higher bioluminescent signals, forming a bioluminescent contour that reflects the actual size of the tumor.

Although this study was limited to the evaluation of mammary tumors, any other tumor that experiences hypoxia should be visible through the use of this system. Thus, this model has the potential for widespread utilization, as it relies upon a characteristic innate to all solid tumors, the presence of relative hypoxia due to poor vascular infiltration of the mass. Thus, an additional option for the non-invasive imaging of a variety of spontaneously occurring tumors is available which will facilitate our ability to gather data in genetically driven mouse cancer models. Moreover, the capability of monitoring the development of the same spontaneous tumors over time makes this model particularly appealing for the *in vivo* evaluation of the efficacy of novel cancer chemotherapeutics.

Drawbacks to this model certainly exist, the key one being the presence of background signals as noted on Figure 2. It is clear that hypoxia is intrinsic in some normal tissues, such as the kidneys and thyroid, which may make the observation or localization of tumors arising near or from these tissues impractical. Moreover, the generally high background observed in the images of the ventral abdomen indicates that tumors located on the dorsal surface of the animal may be more readily imaged than those located elsewhere. Furthermore, our model is also dedicated to the exploration of the mammary carcinoma, an exclusively subcutaneous tumor, and it is difficult to accurately predict what attempts to image deeper tumors may find. Deep tumors likely will emit signals strong enough to be detected, but the distortion of the signals due to travel through heterogeneous tissues will add an additional level of complexity to data interpretation and analysis. Further work will be needed to experimentally determine which types of solid tumors are amenable to this imaging model.

## Materials and Methods

### Ethics Statement

The study involved mice and the protocol had been approved by University of Medicine and Dentistry of New Jersey-Robert Wood Johnson Medical School/Institutional Animal Care and Use Committee (IACUC). Approval ID: I07-038-3. All mice were housed under standard conditions per IACUC and university vivarium protocols in a barrier facility.

### Transgenic Mouse Generation

FVB/N-Tg (MMTV-neu)202Mul/J (referred to herein as *MMTV-neu*) mice, stock number 02376 were obtained from Jackson Laboratories (Bar Harbor, ME) at eight weeks of age and bred with C57BL/6 background *Beclin1* wild-type (+/+) and heterozygous (+/−) mice from a sustained laboratory colony. FVB.129S6-*Gt(ROSA)26Sor^tm1(HIF1A/luc)Kael^*/J (referred to herein as *ODD-Luc*) mice were obtained from Jackson Laboratories at eight weeks of age and cross bred with *Beclin1+/+,MMTV-neu* and *Beclin1+/−,MMTV-neu* mice to generate *Beclin1+/+,MMTV-neu,ODD-Luc* and *Beclin1+/−,MMTV-neu,ODD-Luc* offspring for imaging.

### Mouse Genotyping

Mice were genotyped via tail tip amputation and digestion for DNA extraction utilizing the Qiagen DNeasy assay (Alameda, CA). Following DNA extraction, DNA samples were subjected to polymerase chain reactions with times, temperatures, and DNA primers tailored per existing protocols for the *beclin1* [19], *MMTV-neu*, and *ODD-Luc* genes (available from Jackson Laboratories, www.jaxmice.jax.org). PCR products were loaded into wells in gels consisting of 1.5% agarose, suspended in TSA buffer, and subjected to electrophoresis. The gels were imaged on a UVP Gel Documentation System (Upland, CA) and compared to known controls to determine genotype with respect to genes of interest.

### Mouse Imaging

Mice were selected for imaging and placed in the IVIS induction chamber three at a time and subjected to inhalational isoflurane anesthesia (Abbott, Abbott Park, IL) at 3% with 1 L/min flow of oxygen. Following induction, mice were individually removed from the induction chamber and given an intraperitoneal injection of 50 mg/kg luciferin (Promega, Madison, WI) suspended in sterile phosphate-buffered saline (Invitrogen, Carlsbad, CA). After a ten minute incubation period, the mice were placed on the heated imaging platform of the IVIS Spectrum imaging station (Caliper, Hopkinton, MA) with inhalational isoflurane anesthesia at 1.5% with 1 L/min flow of oxygen during the imaging procedure. White light and luciferase activity images were obtained at 30 second intervals for five minutes. Following imaging, mice were removed from the imaging stage and allowed to recover from anesthesia in a heated cage. Mice were observed for normal behavior prior to being returned to their original housing. Images were subjected to interpretation on Living Image software (Caliper) for evaluation and quantification.

### Immunofluorescence

Paraffin-embedded, slide-mounted tissue sections were deparaffinized and hydrated per standard protocol. Following rehydration, slides were placed in 10 mM Sodium Citrate buffer and placed in a boiling water bath for twenty minutes. Slides were cooled and washing, and tissue sections were blocked for one hour with 10% goat serum in phosphate-buffered saline/Triton X-100 (PBST) in a humid, light-tight box at room temperature. Following blocking, tissue sections were exposed to a 1∶50 dilution of primary anti-firefly luciferase antibody #G7451 (Promega) in PBST overnight. After washing, secondary donkey-anti-goat antibody #SC-2020 (Santa Cruz Biotech, Santa Cruz, CA) was applied at a 1∶100 ratio in PBST with a 1∶10000 dilution of DAPI stain (Invitrogen) for one hour. Slides were then mounted in vectashield medium (Vector Labs, Burlingame, CA) and immediately evaluated for fluorescence on a Zeiss (Thornwood, NY) Axioplan 2 microscope with an EXFO X-cite 120 Fluorescence Imaging System (Quebec, Canada). Digital images were obtained and stored on an Apple (Cupertino, CA) Macintosh G4 computer. Histograms were generated using ImageJ software, freely obtained from the National Institutes of Health at http://rsp.info.nih.gov/ij.

### Mouse Necropsy and Histology

Mice were humanely euthanized via cervical dislocation. Immediately following euthanasia, tumors were dissected out and photographs were taken of the tumor *in situ*. Tumors were then removed, measured, weighed, photographed and sectioned if necessary, and portions were placed in Millonig's 10% buffered formalin (Surgipath, Richmond, IL) for 24 hours prior to being placed in 70% ethanol. Remaining tissue was snap-frozen and stored in liquid nitrogen for future use. Following tumor removal and preservation, a complete necropsy was performed and any gross pathology was noted. Formalinized/ethanol-preserved tissue was submitted to Tissue Analytical Services at the Cancer Institute of New Jersey for paraffin-embedding, sectioning, selective hematoxylin and eosin (H&E) staining, and mounting on glass slides. H&E stained slides were imaged on a Zeiss Axioplan 2 microscope with a Zeiss HAL 100 light source. Digital images were obtained via a Sony Cybershot DSC-HX1 camera mounted with a Micron Optics (Cedar Knolls, NJ) MM99-HX1 microscope adapter.

### Tumor area, tumor volume, radiance calculation, and statistical analysis

Manual measurements of tumor area were obtained by measuring the widest and narrowest axes of the tumor with calipers and then calculating the area of an oval by the following equation: A = π*a*b, where a is the radius of the short axis and b is the radius of the long axis. Computer-obtained cross-sectional tumor areas were obtained with Living Image software (Caliper) through the use of the automatic region of interest (ROI) function. Images were forced to conform to predetermined scales of radiance, and ROIs were automatically generated around tumors with a threshold of 25%, a lower limit of 1, and a minimum size of 20. ROIs were then evaluated for total area and total radiance via the Living Image software, and results were exported to Microsoft Office 2007 (Microsoft, Redmond, WA) for analysis and graphing.

Tumor volume manual measurements were obtained by measuring the widest and narrowest axes of the tumor with calipers from two different views (right lateral and ventral) and then calculating the volume of an ellipsoid by the following equation: V = 4/3 * π * a * b * c, where a is the radius of the x axis, b is the radius of the y axis, and c is the radius of the z axis. Computer-obtained tumor volume was obtained with the Living Image software through the use of the free-style region of interest function. A region of interest was manually drawn around the bioluminescence of the tumor from two different views (right lateral and ventral). Software generated x, y, and z axis radii from the region of interest were used for the volume of an ellipsoid equation.

The size of a palpable tumor in each mouse was measured over the experimental period either manually or by bioluminescence detection. The Pearson correlation coefficient (ρ) was used to assess the correlation between results obtained by the two measurements. All p values are reported as testing the null hypothesis of zero correlation (ρ = ).

### Tumor treatment

Treatment of tumor-bearing animals was conducted as follows: Doxorubicin (4 mg/kg, Sigma) was given every other day for 2 weeks by intraperitoneal injection. In addition, prednisone (0.2 mg/kg, Sigma) was given orally daily for 2 weeks by gavage. Mice were subjected to imaging every other day, starting on day 1 and before each new doxorubicin injection.

## Supporting Information

Table S1 Total Number of F2 (*mmtv-neu/ODD-Luc beclin1+/+ or beclin1+/−*, as described in Figure 1) mice and tumor prevalence in female mice. Normal and tumor bearing female mice were examined in the study.
(DOC)

## References

[1] Weissleder R, Pittet MJ (2008). Imaging in the era of molecular oncology.. Nature.

[2] Kyriazi S, Kaye SB, deSouza NM. Imaging ovarian cancer and peritoneal metastases–current and emerging techniques.. Nat Rev Clin Oncol.

[3] Oyen WJ, van der Graaf WT (2009). Molecular imaging of solid tumors: exploiting the potential.. Nat Rev Clin Oncol.

[4] Waldman AD, Jackson A, Price SJ, Clark CA, Booth TC (2009). Quantitative imaging biomarkers in neuro-oncology.. Nat Rev Clin Oncol.

[5] Dennis C (2006). Cancer: off by a whisker.. Nature.

[6] Frese KK, Tuveson DA (2007). Maximizing mouse cancer models.. Nat Rev Cancer.

[7] Sharpless NE, Depinho RA (2006). The mighty mouse: genetically engineered mouse models in cancer drug development.. Nat Rev Drug Discov.

[8] Ince TA, Richardson AL, Bell GW, Saitoh M, Godar S (2007). Transformation of different human breast epithelial cell types leads to distinct tumor phenotypes.. Cancer Cell.

[9] Schetter AJ, Heegaard NH, Harris CC. Inflammation and cancer: interweaving microRNA, free radical, cytokine and p53 pathways.. Carcinogenesis.

[10] Tuveson DA, Jacks T (2002). Technologically advanced cancer modeling in mice.. Curr Opin Genet Dev.

[11] Jonkers J, Berns A (2002). Conditional mouse models of sporadic cancer.. Nat Rev Cancer.

[12] Gross S, Piwnica-Worms D (2005). Spying on cancer: molecular imaging in vivo with genetically encoded reporters.. Cancer Cell.

[13] Tennant DA, Duran RV, Gottlieb E. Targeting metabolic transformation for cancer therapy.. Nat Rev Cancer.

[14] Kaelin WG (2008). The von Hippel-Lindau tumour suppressor protein: O2 sensing and cancer.. Nat Rev Cancer.

[15] Harris AL (2002). Hypoxia–a key regulatory factor in tumour growth.. Nat Rev Cancer.

[16] Hanahan D, Weinberg RA (2000). The hallmarks of cancer.. Cell.

[17] Safran M, Kim WY, O'Connell F, Flippin L, Gunzler V (2006). Mouse model for noninvasive imaging of HIF prolyl hydroxylase activity: assessment of an oral agent that stimulates erythropoietin production.. Proc Natl Acad Sci U S A.

[18] Muller WJ, Sinn E, Pattengale PK, Wallace R, Leder P (1988). Single-step induction of mammary adenocarcinoma in transgenic mice bearing the activated c-neu oncogene.. Cell.

[19] Yue Z, Jin S, Yang C, Levine AJ, Heintz N (2003). Beclin 1, an autophagy gene essential for early embryonic development, is a haploinsufficient tumor suppressor.. Proc Natl Acad Sci U S A.

[20] Muller WJ (1991). Expression of activated oncogenes in the murine mammary gland: transgenic models for human breast cancer.. Cancer Metastasis Rev.

[21] Muller WJ, Arteaga CL, Muthuswamy SK, Siegel PM, Webster MA (1996). Synergistic interaction of the Neu proto-oncogene product and transforming growth factor alpha in the mammary epithelium of transgenic mice.. Mol Cell Biol.

[22] Stern DF (2008). ERBB3/HER3 and ERBB2/HER2 duet in mammary development and breast cancer.. J Mammary Gland Biol Neoplasia.

[23] Albanell J, Baselga J (1999). The ErbB receptors as targets for breast cancer therapy.. J Mammary Gland Biol Neoplasia.

[24] Dewhirst MW (2009). Relationships between cycling hypoxia, HIF-1, angiogenesis and oxidative stress.. Radiat Res.

[25] Dewhirst MW, Cao Y, Moeller B (2008). Cycling hypoxia and free radicals regulate angiogenesis and radiotherapy response.. Nat Rev Cancer.

[26] Kim JB, Urban K, Cochran E, Lee S, Ang A Non-invasive detection of a small number of bioluminescent cancer cells in vivo.. PLoS One.

