# Supplementary material for: Use of the ODD-Luciferase Transgene for the Non-Invasive Imaging of Spontaneous Tumors in Mice
Source: PLoS One. 2011 Mar 29;6(3):e18269. doi: 10.1371/journal.pone.0018269 (PMC3066234; doi:10.1371/journal.pone.0018269)
Supplement: Table S1 — Total Number of F2 (mmtv-neu/ODD-Luc beclin1+/+ or beclin1+/−, as described in Figure 1) mice and tumor prevalence in female mice. Normal and tumor bearing female mice were examined in the study. (DOC) [file pone.0018269.s001.doc]

**Table S1: Tumor incidence rate in F2 generation of transgenic mice.**

|  |  | |
| --- | --- | --- |
| Total Transgenic Mice | 61 | |
|  | Male | Female |
| By Sex | 30 | 31 |
| Tumor Bearing |  | 11 (29%) |
| Avg Age of Tumor Onset (months) |  | 12.6 |
| Median Age of Tumor Onset (months) |  | 11 |
